# Peripheral Blood Signatures of Lead Exposure

**DOI:** 10.1371/journal.pone.0023043

**Published:** 2011-08-01

**Authors:** Heather G. LaBreche, Sarah K. Meadows, Joseph R. Nevins, John P. Chute

**Affiliations:** 1 Duke Institute for Genome Sciences and Policy, Duke University, Durham, North Carolina, United States of America; 2 Department of Molecular Genetics and Microbiology, Duke University, Durham, North Carolina, United States of America; 3 Program in Genetics and Genomics, Duke University, Durham, North Carolina, United States of America; 4 Division of Cellular Therapy, Department of Medicine, Duke University, Durham, North Carolina, United States of America; 5 Department of Pharmacology and Cancer Biology, Duke University, Durham, North Carolina, United States of America; East Carolina University, United States of America

## Abstract

**Background:**

Current evidence indicates that even low-level lead (Pb) exposure can have detrimental effects, especially in children. We tested the hypothesis that Pb exposure alters gene expression patterns in peripheral blood cells and that these changes reflect dose-specific alterations in the activity of particular pathways.

**Methodology/Principal Finding:**

Using Affymetrix Mouse Genome 430 2.0 arrays, we examined gene expression changes in the peripheral blood of female Balb/c mice following exposure to *per os* lead acetate trihydrate or plain drinking water for two weeks and after a two-week recovery period. Data sets were RMA-normalized and dose-specific signatures were generated using established methods of supervised classification and binary regression. Pathway activity was analyzed using the ScoreSignatures module from GenePattern.

**Conclusions/Significance:**

The low-level Pb signature was 93% sensitive and 100% specific in classifying samples a leave-one-out crossvalidation. The high-level Pb signature demonstrated 100% sensitivity and specificity in the leave-one-out crossvalidation. These two signatures exhibited dose-specificity in their ability to predict Pb exposure and had little overlap in terms of constituent genes. The signatures also seemed to reflect current levels of Pb exposure rather than past exposure. Finally, the two doses showed differential activation of cellular pathways. Low-level Pb exposure increased activity of the interferon-gamma pathway, whereas high-level Pb exposure increased activity of the E2F1 pathway.

## Introduction

While substantial progress has been made in identifying the alterations in cancer genomes, much less is understood of the contributions of environmental agents in defining the course of cancer development. Mounting evidence indicates a role for lead in carcinogenesis. Epidemiological studies show that environmental and occupational lead exposures increase cancer risk, particularly lung and stomach cancer [1]–[4]. There is also ample evidence from animal studies that lead is carcinogenic, causing lung, brain, hematopoietic and kidney tumors [5]–[7]. Furthermore, recent studies have revealed several biological mechanisms that might contribute to the carcinogenic effects of lead, including the inhibition of DNA synthesis and repair, oxidative damage, interaction with DNA-binding proteins and tumor suppressor proteins and alterations to gene transcription [6]–[9]. Lead has also been shown to damage chromosomes by causing micronuclei formation, chromosomal aberrations and sister chromatid exchanges. [6], [10]. Finally, lead enhances the carcinogenicity of other genotoxic and mutagenic substances such as UV-radiation, oxygen radicals and various chemical mutagens in the context of co-exposure [11]–[15]. Therefore, lead exposure can cause the deregulation of several cellular pathways that are critical for normal cell division, DNA synthesis, DNA and damage repair and gene transcription, setting the stage for tumorigenesis.

An ability to detect and quantify the effects of past and present lead exposure on specific cellular pathways might facilitate the monitoring of individuals for increased cancer risk and also contribute to an understanding of how lead exposure contributes to carcinogenesis. Current research emphasizes the need to evaluate the mechanisms of lead-induced carcinogenesis, particularly oxidative stress/apoptosis and the roles of cellular defense mechanisms, signaling pathways, and intracellular lead-binding patterns.

Because children are especially susceptible to the effects of lead, the Center for Disease Control and Prevention (CDC) has led nationwide efforts to eliminate lead exposure in children. Although Pb has been banned from gasoline, residential paint and solder used for food cans and water pipes, the CDC estimates that over 300,000 U.S. children (ages 1–5 years) have elevated blood lead levels (BLLs) [16]. Exposure is most likely to occur through inhalation or ingestion of Pb dust or through exposure to soil or water contaminated with Pb from industrial and manufacturing sources. Pb poisoning in children can cause brain damage, behavioral problems, growth delays, hearing problems, headaches and in rare cases seizures, coma and even death [6]. The CDC has set a goal to eliminate BLLs ≥10 µg/dL in young children by 2010. However, many studies indicate that even BLLs below 10 µg/dL can cause negative health effects [17]–[19] and the CDC has stated that there is “no safe blood lead level” in children [16].

Accumulating evidence indicates that low-level Pb exposure can have a detrimental effect on health, especially that of children. In addition, many studies point to a role for Pb in facilitating carcinogenesis. Therefore, sensitive tools are needed to be able to detect past and present Pb exposures, even at low levels. In addition to assessing individual exposure history, it is imperative to be able to understand the biological mechanisms through which Pb contributes to negative health outcomes, including increased cancer risks. Therefore, two major challenges in the field of Pb toxicology are to 1) evaluate the health impact of low-level exposures that do not cause any observable health effects and 2) understand the mechanisms by which Pb affects normal cellular pathways and how this may contribute to future health risks.

Many groups are applying genomic tools to the study of Pb toxicology. For example, Ruden et al. used expression quantitative trait locus mapping techniques to identify genomic regions containing putative “master-regulators” of response to Pb exposure in fruit flies [20]. Bouton et al. [21] characterized gene expression in immortalized astrocytes, thus confirming several genes previously reported to be induced by lead and also identifying some novel ones. This work prompted a follow up study, which investigated the mechanisms by which Pb induced vascular endothelial growth factor (VEGF) expression in astrocytes, demonstrating the potential for microarray studies to reveal novel mechanisms of toxicity [22]. Finally, Kasten-Jolly et al. have described the effects of developmental Pb exposure on gene expression in the spleen [23]. The results of this study provided insight into the role of Pb exposure on inhibition of heme biosynthesis and the generation of peptides that could contribute to autoimmune syndromes. It is vital to understand the effects of Pb exposure on target tissues and organs, and the gene expression patterns described in these studies serve as biomarkers of effect. However, many of the tissues known to be affected by Pb (e.g. spleen and brain tissues) are not easily accessible and thus do not represent a means to assess exposure in a noninvasive manner. Our study, which presents the first microarray analysis of the effects of Pb exposure on PBCs, highlights the potential to use blood-based biomarkers to identify low- and high-level exposures and gain a better understanding of the underlying mechanisms of Pb toxicity.

Previous studies have highlighted the opportunity to use gene expression patterns in peripheral blood cells (PBCs) as a basis for measuring exposure to environmental agents as well as a way to probe the underlying mechanisms of toxicity. These studies capitalize on the natural facility of PBCs as indicators of environmental exposure combined with the power of global gene expression analysis. For example, molecular signatures generated to diagnose radiation exposure [24]–[26] also have the potential to identify those genes that are affected by radiation exposure and may have implications in the treatment of radiation injury. Many other groups have described blood-based gene expression signatures of occupational and environmental exposures [27]–[30].

We use a similar approach to develop signatures of Pb exposure based on peripheral blood cell gene expression. However, our approach is unique in that we have made use of a collection of signatures that represent the activation of various cell-signaling pathways to identify the pathways that are either activated or repressed in response to Pb exposure. We describe dose-specific signatures of Pb exposure in a mouse model and also identify pathways that are perturbed in a dose-specific manner. These findings suggest that the response to Pb exposure may be quite distinct depending on the level of exposure. In addition, our results confirm previous studies that suggest there is no safe level of Pb exposure. Even low levels of Pb exposure alter normal gene expression patterns in peripheral blood cells and result in the aberrant activation of cellular signaling pathways.

## Results

**Figure 1:**
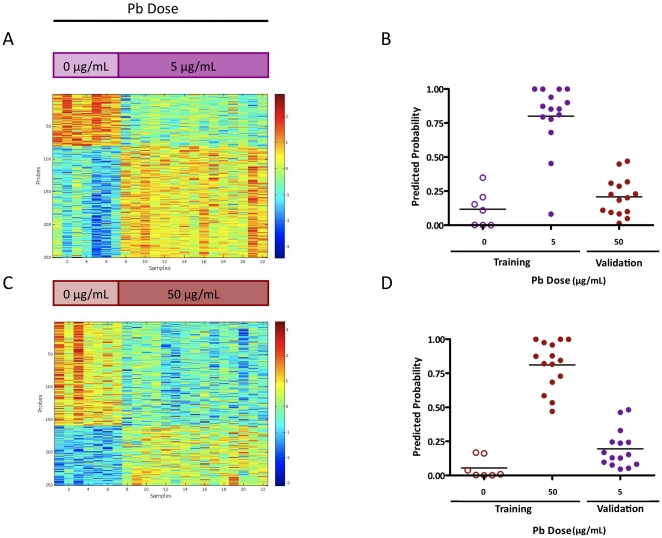
Dose-Specific peripheral blood signatures. We generated signatures of lead exposure based on gene expression analysis of whole blood from exposed mice and control mice. A) A 250-probe signature of low-dose lead exposure is shown as a heatmap, where each column represents an individual biological sample and each row represents an Affymetrix M430 2.0 probe identifier. Red =  high expression; blue  =  low expression. B) The signature is able to accurately distinguish samples from control mice from those exposed to low levels of lead as shown in the leave-one-out crossvalidation. However, this signature is specific to low level lead exposure, as there is no significant difference in predicted probabilities of samples from control mice and those exposed to high-dose lead. C) A 250-probe signature of high-dose lead exposure is shown as a heatmap of gene expression. D) While the signature is able to accurately distinguish between samples from control mice and mice exposed to high levels of lead, it is unable to classify samples from mice exposed to low lead levels, demonstrating its dose specificity. Open purple circles  =  control mice; close purple circles  =  mice exposed to low-level lead; open red circles  =  control mice; close red circles  =  mice exposed to high-dose lead.

We generated blood-based gene expression signatures that reflect changes in the transcriptome of peripheral blood cells. As shown in Figure 1, we were able to generate robust signatures for each dose of Pb exposure. Each signature demonstrated a strong capacity to distinguish Pb-exposed mice from controls in a leave-one-out crossvalidation. The low Pb signature (Figure 1A–B) showed a sensitivity of 93% and specificity of 100% based on a receiver-operator characteristic curve (AUC = 0.9619, p-value = 0.0006340). The high Pb signature (Figure 1C–D) showed 100% sensitivity and specificity in classifying samples based on exposure (AUC = 1.000, p-value = 0.0002173). It was also evident from this analysis that the signatures were dose-specific. In other words, the high dose signature could not accurately distinguish low Pb exposure from control and *vice versa* (Figure 1B, D). Consistent with this specificity, the 250-probe signatures only have 28 probes in common. (Table S1 lists the probes in each signature along with the corresponding gene symbol and description.)

**Figure 2:**
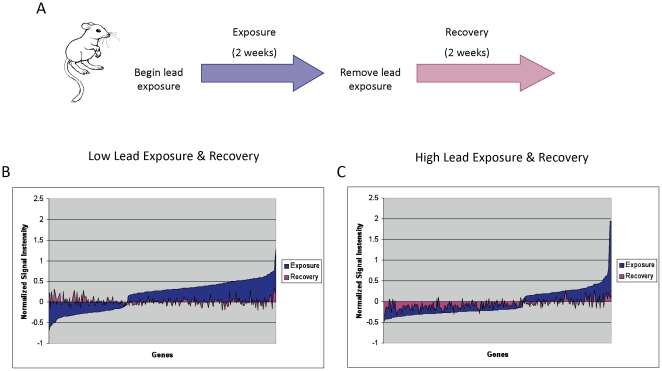
Transcriptional changes following exposure and recovery. **A**) Schematic of experimental design, in which mice were exposed to lead *via* drinking water over the course of two weeks at which time a blood sample was collected. Then the lead source was removed and the mice had a two-week recovery period before a second blood collection. **B**) The x-axis represents the 250 probes in the low-dose lead signature, ordered from lowest to highest average expression value relative to controls. The y-axis represents the normalized average signal intensity of the probe across the samples. The blue field represents the mean signal intensity of the signature probes in mice exposed to low-dose lead, normalized by subtracting the mean values in the control mice. The magenta field represents the mean signal intensity of the same signature probes after the two-week recovery period and normalized against controls. **C**) A comparison of the gene expression signature in mice following two weeks of exposure to high-dose lead (blue) and then two weeks of recovery (magenta).

A focus of the approach of developing gene expression signatures of Pb exposure was the potential that this might provide a method sensitive enough to reveal a history of exposure. To evaluate the extent to which the signature persists following Pb exposure, we analyzed the expression of the signature genes following a two-week absence of Pb in the drinking water. As shown in Figure 2, the effect was transient, with the expression of the signature genes returning to the level of background after two weeks of Pb-free drinking water. As such, it would appear that the distinct expression signature resulting from Pb exposure reflects the acute and current exposure to Pb rather than a lasting effect on gene transcription patterns in the peripheral blood cells. The normalized gene expression value for the genes in each signature relative to control values is listed in Table S2.

**Figure 3:**
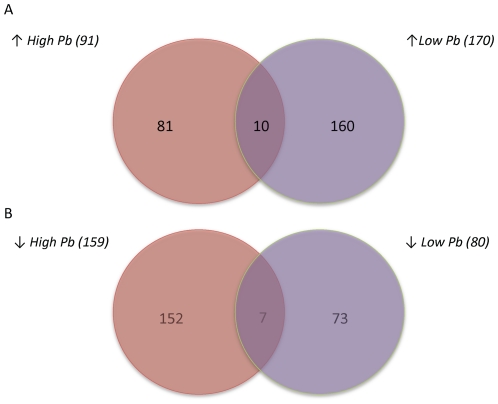
Overlap between upregulated and downregulated genes. Each signature was broken down into genes either upregulated or downregulated in response to lead exposure compared to controls. **A**) A Venn diagram comparing the upregulated gene lists from each signature (high-dose lead, n = 91; low-dose lead, n = 170). We identified 10 overlapping upregulated genes. **B**) By comparing those genes downregulated in response to each dose of lead, we identified 7 overlapping genes (high-dose lead, n = 159; low-dose lead, n = 80).

In order to better understand the similarities and differences between the two signatures, we broke each signature into two components – genes that were either upregulated or downregulated in response to Pb exposure – and compared these for each dose. The high dose Pb signature was composed of 91 upregulated probes and 159 downregulated probes. The low dose Pb signature was composed of 170 upregulated probes and 80 downregulated probes. A comparison of the upregulated probes revealed that the signatures shared only 10 in common; a comparison of the downregulated probes revealed that the signatures only shared 7 in common (see Figure 3). Table S3 shows the top gene ontology categories for the genes that are upregulated and downregulated in response to each level of Pb exposure. An analysis of the gene ontology categories that were significantly enriched in the upregulated sets of genes revealed that although the genes induced by the low and high doses of Pb did not overlap extensively, they reflected similar biological processes, namely the cellular response to unfolded proteins. However, the high-dose Pb signature also includes several upregulated genes related to the processes of angiogenesis and blood vessel formation. A similar analysis of the gene ontology categories enriched in the downregulated genes revealed that the high dose signature genes were related to the cellular response to heat, and G-protein coupled receptor signaling. The downregulated genes within the low dose signature represented anti-apoptotic genes and genes involved in antigen presentation. A particularly interesting finding came from a comparison of those genes downregulated in response to high levels of Pb, but upregulated in response to low doses. This particular subset of genes was enriched for binding sites for various hormone receptors, such as androgen receptor, progesterone receptor and glucocorticoid receptor (Table S4).

**Figure 4:**
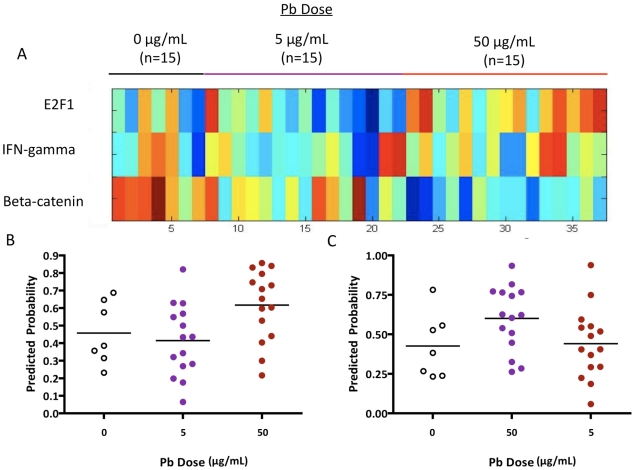
Differential pathway activation. **A**) A heatmap displaying the predictions of pathway activity shows the predicted probability of activation of a particular pathway (E2F1, IFN-gamma) across samples from mice exposed to different levels of lead. Red =  high probability; blue  =  low probability. **B**) Scatter plot of predicted E2F1 pathway activity for samples from mice exposed to low-dose lead (purple circles), high-dose lead (red circles) or no lead (control, open circles). The x-axis represents the exposure group and the y-axis represents the probability that the E2F1 pathway is activated in an individual sample. **C**) Predicted probabilities for IFN-gamma pathway activity in mouse blood samples.

In light of the differences we observed between the low-dose and high-dose Pb signatures, we hypothesized that Pb exposure could result in differential activation or repression of cellular pathways based on the level of exposure. To test this theory, we took advantage of a collection of gene expression signatures representing the activity of particular cellular pathways [31], [32]. We applied this set of signatures to the gene expression data from Pb-exposed mice and looked for pathways that were differentially regulated based on level of exposure. As shown in Figure 4, the E2F1 pathway was found to be upregulated in the blood of mice exposed to high doses of Pb relative to the low dose (p-value 0.0249, F-value  = 4.126). We also observed a trend in which the interferon-gamma (INFγ) pathway was upregulated in the blood of mice exposed to low doses of Pb as compared to the high dose (p-value  = 0.0840, F-value  = 2.667).

## Discussion

The concept of using the blood to assess Pb exposure is well-established in the medical and public health fields. Measuring BLLs directly is the most common method of determining blood Pb concentration. BLL is a measure of circulating Pb and does not measure total Pb stored in the body. Nor does it measure the effects of current or cumulative Pb exposure. Pb is also known to impair heme biosynthesis. Therefore, an alternative method to screen for Pb exposure is to measure the level of erythrocyte protoporphyrin (EPP) or zinc protoporphyrin (ZPP), which is an early and reliable indicator of impaired heme biosynthesis. This method is not sensitive enough to be used to screen children, but can be used in monitoring occupationally exposed adults and can reflect average Pb exposure over a period of a few weeks. Our approach, as outlined in Figure 1, was to measure global gene expression in the blood of mice exposed to lead acetate via their drinking water and to generate a signature based on the genes most highly correlated with the exposure. Our hypotheses were that 1) PBC gene expression signatures would be good indicators of low-level Pb exposure which is not associated with observable effects, but contributes to negative health outcomes, and 2) PBC gene expression signatures may reflect past exposures, representing an opportunity to characterize an individual’s exposure history and its relationship to current health issues and future health risks.

We report robust and dose-specific blood-based signatures of Pb exposure. The low-level Pb signature is particularly promising because it represents an estimated BLL of 3.3 µg/dL, which is well below the 10 µg/dL level for children set by the CDC. It is also below the lower limits of detection for the EPP and ZPP tests. The ability to detect such low-level Pb exposure could allow researchers to better monitor low-level exposures and associated side effects (both short-term and long-term).

Unfortunately, the signatures reported here do not seem to be indicators of past exposure. However, the gene expression data generated provide a wealth of molecular information that is useful in understanding the biological processes and signaling pathways underlying the toxic effects of Pb at different doses. Many toxicogenomic studies have analyzed transcriptome or gene expression data using various annotation or pathway building tools. We have used similar approaches to confirm some important genes and pathways that have previously been implicated in Pb exposure, such as the unfolded protein response, cell death processes, angiogenesis and endocrine disruption. We observed the upregulation of genes related to the unfolded protein response in both the low and high Pb signatures. The unfolded protein response (UPR) involves the transcription of chaperones used to aid in proper protein folding and is known to be triggered by some toxicants, including Pb, whichis thought to trigger the UPR through a mechanism that involves inhibiting protein folding by substituting for zinc or calcium ions [31]. Apoptotic cell death is known to play a role in lead-induced neurotoxicity [33]–[35], and it is feasible that it occurs in the peripheral blood even at low levels of exposure. Additional anti-apoptosis genes are actually downregulated in the low Pb signature, strengthening the argument that this low level of exposure induces cell death in the PBCs. The high Pb signature also included some upregulated genes related to angiogenesis and blood vessel formation. This is consistent with work mentioned previously, in which the mechanism of Pb-induced VEGF expression was elucidated in astrocytes [21], [22]. Interestingly, a subset of genes that were upregulated in response to low doses of Pb and downregulated in response to high doses of Pb was significantly enriched for binding sites for various hormone receptors, such as androgen, progesterone and glucocorticoid receptors. Although Pb is a known endocrine disruptor, leading to reproductive impairment in vertebrates, the mechanisms for this are not entirely clear and the effects of low-level exposure are not well understood [36]–[39].

In addition to traditional gene annotation approaches, we also use a novel approach to assess pathway activity by applying a panel of gene expression signatures generated by specific perturbations in controlled in vitro experiments to our dataset. Although use of this panel of signatures is not new, its application to evaluating toxicant exposure is. This approach allows us to assess the relative activity of a given pathway across samples. This approach has been successful in identifying the deregulation of oncogenic pathways in either cancer cell lines or patient tumor samples and predicting which targeted therapies might be most effective [32], [40]. In the context of environmental exposure to toxic metals, this approach could be used as a tool for discovery to identify new pathways that are affected by a particular toxicant or as a means of studying how pathway activity varies depending on factors, such as level of exposure, age, sex and co-exposure with other toxicants.

Our findings suggest that distinct biological processes are involved in the response to Pb depending on the level of exposure. Unlike the current methods used to assess Pb exposure, this approach not only assesses exposure in a dose-specific manner but it also provides information about the effects of the exposure on cellular pathways and processes. At low levels of exposure, the IFNγ pathway is upregulated. This supports previous findings that Pb inhibits IFNγ production in a dose dependent fashion [41] enhancing development of Th2 (Type 2 T helper cell) responses versus Th1 (Type 1 T helper cell) responses [23], [37]. Mechanistic studies suggest that Pb selectively inhibits translation of IFNγ [42]. IFNγ plays a major role in immune system function by regulating macrophage activation, differentiation of progenitor helper T cells and enhancement of the major histocompatibility complex molecule expression. INFγ is known to have some immunomodulatory effects and may be responsible for the disruption to the various endocrine pathways observed in response to low-dose Pb exposure. It is responsible for regulating cell-mediated immune responses to infectious pathogens. It is also reported to have antitumor activity. Even transient disruption of this pathway in response to low levels of Pb exposure may have significant consequences in terms of susceptibility to disease, including infection, allergy and even cancer.

High Pb levels were found to enhance E2F1 pathway activity. This is also supported by previous findings that suggest that Pb exposure affects cell cycle and can actually increase DNA synthesis. This has been observed in a variety of contexts. Pb stimulated DNA synthesis in vascular smooth muscle cells [43] and rat kidney cells [44]. Razani-Boroujerdi et al. [45] demonstrated that Pb enhanced proliferation of rat splenic lymphocytes *in vitro.* Furthermore, Lu et al. [46] has shown that Pb stimulates DNA synthesis in human astrocytoma cells. Increases in cell proliferation have also been observed *in vivo* in both rat kidney [47] and rat liver [12], [48]. This cumulative evidence for the role of Pb in enhancing cell proliferation and DNA synthesis may have relevance to the potential role of Pb in carcinogenesis.

Our work has demonstrated the ability to detect the effects of Pb exposure on PBC gene expression, even at low levels of exposure. Furthermore, we have shown that the IFNγ and E2F1 pathways are aberrantly regulated in response to low-level and high-level Pb exposure respectively. These results underscore the importance of understanding the effects of Pb exposure on critical cellular pathways. By identifying the pathways that are impacted by Pb, we can better understand the mechanisms underlying the negative health consequences of Pb exposure and gain insight into the role of environmental and occupational exposures in contributing to overall health.

## Materials and Methods

### Ethics Statement

Animal use and husbandry was conducted humanely and with regard to alleviation of suffering in accordance with the Guide for the Care and Use of Laboratory Animals and all procedures were approved by Duke University Institutional Animal Care and Use Committee (IACUC) under protocol number A122-08-05.

### Murine Pb Exposure Study

Nine-week old female Balb/c mice (Jackson Laboratories, Bar Harbor, ME) were divided into three groups. All mice received Pb-free rodent chow. Each group received drinking water with different concentrations of Pb in the form of lead acetate trihydrate (Sigma Aldrich, St. Louis, MO) for a period of two weeks. The control group (n = 14) received 0 µg/mL; the low exposure group (n = 15) received µg/mL; and the high exposure group (n = 15) received 50 µg/mL. We chose these concentrations of Pb based on previous studies by Iavicoli et al. [49], who found that the relationship between the amount of Pb administered in the drinking water and the resulting BLL after two weeks of exposure could be represented by the following formula:
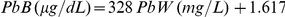



In this formula PbB is blood-lead concentration and PbW is water-lead concentration [49]. Using this equation, we estimate the final PbB concentration in the mice to be approximately 3.3 µg/dL in the low-level exposure group 18 µg/uL in the high-level exposure group. These levels would be in addition to any background levels of Pb due to contamination of the drinking water. After two weeks of exposure, peripheral blood was collected by submandibular bleed from half of the control mice (n = 7) and all of the exposed mice (n = 15 per group). All mice were then switched to Pb-free drinking water for two additional weeks. A second blood sample was collected after this recovery period (controls, n = 7; low Pb, n = 15; high Pb, n = 13). In the high Pb group, 1 mouse died during the recovery period, and 1 blood sample was not used due to contamination. All blood samples (72 total blood samples) were collected using GoldenRod animal lancets (MEDIpoint, Inc., Mineola, NY) and stabilized using RNAProtect Animal Blood Tubes and total RNA was extracted using the RNEasy Protect Animal Blood Kit (Qiagen, Valencia, CA). Globin transcript was removed using GLOBINclear for mice (Ambion, Austin, TX).

### RNA Preparation and Microarray Analysis

Total RNA quality was assessed using an Agilent 2100 Bioanalyzer (Agilent, Palo Alto, CA). Each sample (150 ng) was amplified using the MessageAmp Premier RNA Amplification Kit (Ambion, Austin, TX) and analyzed using the Mouse Genome 430 2.0 Array (Affymetrix, Santa Clara, CA). The data discussed in this publication are MIAME compliant and have been deposited in NCBI's Gene Expression Omnibus (GEO). The are accessible through GEO Series accession number GSE28261 (http://www.ncbi.nlm.nih.gov/geo/query/acc.cgi?acc=GSE28261).

### Statistical analysis

We compared gene expression profiles from whole blood samples isolated from mice exposed to lead acetate through drinking water or control mice. We generated a Pb exposure-associated gene expression signature composed of a subset of genes most correlated to Pb exposure by applying established methods of supervised classification and binary regression [50] to data normalized using the Robust Multi-array (RMA) method. The data presented here are based on signatures composed of 250 Affymetrix probes for the purposes of comparing gene lists of equivalent size. Optimal signatures for each dose (meaning the signature used to achieve maximum accuracy in a leave-one-out crossvalidation) may vary from those reported here. We calculated the average probe expression value for each group of mice at each timepoint (following exposure and recovery) and calculated the level of gene expression as the difference between Pb-exposed mice and controls for each timepoint.

### Gene Set Enrichment Analysis

Gene Set Enrichment Analysis (GSEA, version 2.0.5) was used as previously described [51], [52]. Briefly, we used the gene set annotation feature of GSEA (http://www.broadinstitute.org/gsea/msigdb/annotate.jsp) to identify those gene sets that overlap with the Pb signatures. These gene sets offer insights into the functional annotation of our experimentally derived signatures and have the potential to reveal the underlying biology of each signature. First, we copied the list of the Affymetrix probe identifiers comprising each signature generated by the binary regression algorithm (genecoefficients.txt) and pasted it into the browser’s query field. After selecting the appropriate identifier platform (Affymetrix M430 2.0), we chose to compute overlaps between each signature and the following: C2, or curated gene sets, which is a collection of 1,892 curated gene sets; TFT, or transcription factor targets, which includes 500 gene sets that contain genes that share a transcription factor binding site defined in the TRANSFAC (version 7.4, http://www.gene-regulation.com/) database; and C5, which are gene sets that are named by GO term and contain genes annotated by that term. We focused on the top 20 gene sets in our efforts to annotate the biological function of each signature.

### Signature Annotation

In order to better understand the underlying biological response represented by each Pb exposure signature, we used publicly available tools to annotate each signature. Briefly, each signature can be represented by a list of Affymetrix (M430 2.0 Array) probe identifiers. This list was used as the input into GATHER (http://gather.genome.duke.edu/) to generate the following annotations: gene ontology (GO); KEGG (Kyoto Encyclopedia of Genes and Genomes) pathways; and enrichment for transcription factor binding sites (TRANSFAC v8.2 Professional). We also used the Affymetrix NetAffx online batch query tool to translate each factor from its list of M430 2.0 Array probe identifiers to gene symbols and descriptions (https://www.affymetrix.com/analysis/netaffx/index.affx).

### Pathway Analysis

We analyzed the activity of several experimentally derived pathway signatures as they related to our Pb exposure dataset. Briefly, we used the ScoreSignatures module found on GenePattern (https://cagt.igsp.duke.edu/gp/) to score a series of signatures on our gene expression dataset. First, the RMA-normalized data were translated from Affymetrix M430 2.0 probe identifiers to HU133 Plus 2.0 using the FileMerger tool (http://filemerger.genome.duke.edu/) and a bridging file from ChipComparer (http://chipcomparer.genome.duke.edu/). We averaged gene expression values for duplicated probes using an R script (by Jeff Chang, see Supplemental Materials). The dataset file was then saved in gct file format and uploaded to the GenePattern server. After running the ScoreSignatures module, we plotted the predicted pathway probabilities (probabilities.txt) for each pathway in GraphPad Prism version 4.0b (GraphPad Software, Inc., La Jolla, CA). We tested whether the predicted probabilities for each class were significantly different using a One-way ANOVA, with Bonferroni’s Multiple Comparison Test to compare the classes in a pairwise fashion.

## Supporting Information

Table S1 Affymetrix HU133 Plus 2.0 probe identifiers from each Pb signature and corresponding gene symbol, gene title and GO biological process term.
(XLS)

Table S2 Normalized average signal intensity of all probes comprising each signature. The Exposure column reflects gene expression following 2 weeks of Pb exposure. The values represent the mean signal intensity (RMA-normalized) across all samples from the exposure group minus the mean signal intensity of the samples from the control group. Likewise, the Recovery column reflects gene expression following 2 weeks of Pb-free water. The values represent the mean signal intensity of the samples from the Pb-exposed mice minus the mean signal intensity of the samples from the control mice.
(XLS)

Table S3 GO annotation terms for upregulated and downregulated genes in both high- and low-level Pb signatures.
(XLS)

Table S4 TRANSFAC annotations for those genes that are upregulated in response to low-level Pb exposure, but downregulated in response to high-level Pb exposure.
(XLS)
